# Effects of tranexamic acid on the recovery of osteochondral defects treated by microfracture and acellular matrix scaffold: an experimental study

**DOI:** 10.1186/s13018-019-1144-7

**Published:** 2019-04-15

**Authors:** Erdem Degirmenci, Kutay Engin Ozturan, Abdullah Alper Sahin, Fahri Yilmaz, Yasin Emre Kaya

**Affiliations:** 1 Faculty of Medicine, Department of Orthopaedic and Traumatology, Duzce University, Duzce, Turkey; 2 Faculty of Medicine, Department of Orthopaedic and Traumatology, Abant Izzet Baysal University, Bolu, Turkey; 3 Department of Orthopaedic and Traumatology, Tokat Turhal State Hospital, Tokat, Turkey; 4 Faculty of Medicine, Department of Pathology, Sakarya University, Sakarya, Turkey

**Keywords:** Osteochondral defect, Microfracture, A-cellular scaffold, Tranexamic acid

## Abstract

**Background:**

Microfracture and scaffold application in the treatment of osteochondral defects is still one of the most frequently used methods in the clinic. The most important step in this treatment method is the stabilization of fibrin clot. Tranexamic acid (TA) is an antifibrinolytic agent commonly used in orthopedic surgery in recent years. This study evaluated the effect of local TA application on healing of experimentally induced osteochondral defects on rabbits.

**Methods:**

This paper contains an animal in vivo data and histological outcomes on the effect of TA. Eighteen New Zealand white rabbits were treated unilaterally and cylindrical defects having a width of 4 mm and depth of 5 mm were created in the weight-bearing surfaces of the medial and lateral condyles of the right femur. They were divided into two groups, as group 1 study and group 2 control groups, respectively. One milliliter (ml) of TA was injected into the knee joints of the subjects in group 1. All animals were sacrificed for the extraction of the femur condyles for histologic study at the fourth and eighth weeks after surgery. Histological evaluations were performed by Brittberg and O’Driscoll scores to all samples. Data were organized in a Standard Statistical Package System v.22 software package (SPSS/PC Inc., Chicago, IL.) and reported as mean and median (min-max). Repeated measures ANOVA test was used to compare groups and condyle effects together for each week. *p* values below 0.05 were considered as statistically significant.

**Results:**

Samples were taken in the fourth and eighth weeks. The regularity of the surface in group 1 was smoother, and the tissue stability was more robust. Mean Brittberg scores in both weeks were statistically higher in group 1 when compared with group 2. In the microscopic evaluation, it was observed that the regeneration of subchondral and cartilage tissues were more rapid and organized in group 1, and the mean O’ Driscoll scores in both weeks were statistically higher in group 1.

**Conclusions:**

Application of TA improves the healing time and tissue stability in osteochondral defects which are implanted a-cellular scaffold after microfracture and should be applicable to humans for the treatment of osteochondral defects.

**Electronic supplementary material:**

The online version of this article (10.1186/s13018-019-1144-7) contains supplementary material, which is available to authorized users.

## Background

Recent improvements in the field of science and medicine during the last decade lead to a prolongation of lifespan and increase the importance of sports in human life. However, the frequency of traumatic and degenerative cartilage injuries increases, making the treatment strategies more important in the meantime. Untreated cartilage and osteochondral injuries resulting with severe pain, restricted movements, and deterioration in the quality of life may proceed to diffuse intracartilage damage and osteoarthritis in which major surgical procedures are mandatory for treatment [1].

Intrinsic activity and regeneration ability of cartilage tissue is extremely limited [2]. Cellular migration to this area is restricted due to the density and the avascularity of the tissue. Thus, there is a lack of cell and mediator transfer from adjacent subchondral and synovial tissues to the damaged area, leading to an insufficient recovery of the isolated cartilage and osteochondral tissues [3].

Methods that are used for the recovery of the cartilage tissue can be summarized as microfracture and scaffold procedures, progenitor cell-seeded scaffold procedures, otolog chondrocyte culture, and implantations [4]. However, there are further studies ongoing to obtain the original hyaline cartilage tissue. The most common method in clinical practice is the microfracture and scaffold implantation procedures due to its favorable cost and successful results of the experimental studies [4].

Materials that are used in the experimental design of scaffolds include collagen, hyaluronic acid (HA), polylactide, fibrinogen, and hydroxyapatite which are bioabsorbable [5]. Hyaluronic acid-based biomaterials are the most favorable materials due to their ability to bind CD44 surface receptors of chondrocytes or mesenchymal stem cells. Moreover, they are responsible for the production of the extracellular matrix of the direct chondrogenic genes [6]. Various biomaterials such as natural polymers like collagen, agarose, and hyaluronan or synthetic polymers like polylactic acid, polyglycolic acid, or the components of both are experimentally or clinically in use, still [7–9]. Hyaluronan is a non-immunogenic, biocompatible, and soluble macromolecular polysaccharide which is formed by the repetition of *N*-acetyl-D-glucosamine and D-glucuronic units. Non-crosslinked scaffolds are more soluble due to their affinity to water and their breakage via hyaluronidase enzyme [10].

The success of this procedure can be attributed to the stability of the clot on the scaffold, and its suitability as a host for the progenitor cells arising from subchondral tissue following microfracture, leading to an improvement of regenerating tissue [11–13].

Tranexamic acid is a synthetic antifibrinolytic agent in the stabilization of fibrin via inhibiting the plasminogen activation which is very popular in orthopedic surgery by decreasing the amount of bleeding after major surgeries [14, 15]. It can both be applied topically and by intravenous route. However, topical usage is more favorable due to the possible side effects and safety issues of intravenous administration [16].

Regarding our literature knowledge about TA as a fibrin stabilizer, we aimed to investigate the effects of intraarticular TA application on the recovery of experimental osteochondral lesions following microfracture and a-cellular scaffold application.

## Methods

This study was carried out with the approval of the local ethics committee of animal research of Abant Izzet Baysal University (AIBU) with the decision numbered 08.03.2017/11. European laws on animal experimentation were strictly followed throughout the study, and the animal experimental protocol was approved as requested by Turkey Law according to EC rules (Law by Decree, 2010/63/EU). The popular method in cartilage repair techniques using biomaterials with small experimental animals is to create a defect in femoral condyles and trochlea. Rabbits represent a species, which is very suitable to test newly advanced biomaterials or new therapies as they can be handled easily, are relatively inexpensive, and offer a good joint size for surgical procedures. Larger animals, like, e.g., sheep or goats, are relatively more expensive and reasonably considered to be used in later preclinical studies [17]. New Zealand white rabbit is a type that is suitable for the durability tests of the traditional regulatory committee and is recommended in the ISO 10993 guidelines. Eighteen rabbits weighing 2500 g for 4 months were obtained from the experimental animal care center of AIBU with the support of Duzce University Scientific Research Projects Support Fund. Production and growing of animals and experimental study were made in the same center. The animals were maintained at 20 ± 3 °C with a relative humidity of 40–60% and a photoperiod of 12/12 h, light and dark. Each rabbit was housed in a cage of stainless steel with the bottom grid. The animals were fed with a pellet diet and had access to tap water continuously.

### Surgical procedure

**Fig. 1:**
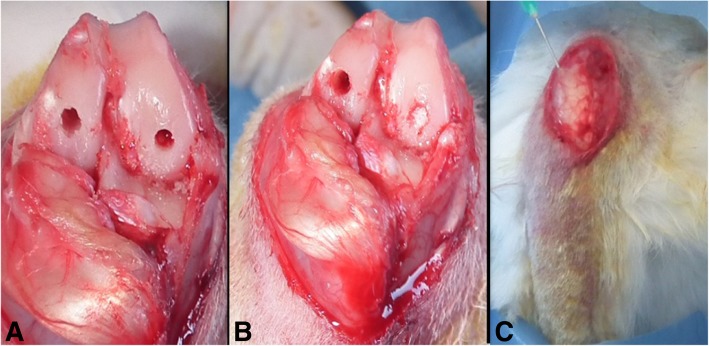
Surgical method. **a** Bicondylar cylindrical osteochondral defect. **b** A-cellular scaffold application to medial femoral condyle. **c** Intraarticular TA application

All animals were treated unilaterally. The right knees of the rabbits were shaved, and the surgical area was removed from the hair and cleaned with betadine and closed with surgical dressing. After the skin incision, a 3-cm medial parapatellar incision was applied and the patella was everted. Cylindrical osteochondral defects were created at the weight-bearing surfaces of the medial and lateral condyles in the size of 4 mm wide and 5 mm deep, by using burr. Guidelines of the American Society for Testing and Materials International indicate that the critical size of a chondral defect in a rabbit is 3 mm in diameter [18]. Kirschner wire was used to induce a microfracture on all defects. Consequently, 36 cylindrical osteochondral defects were created on the condyles. Transposition of the clot from subchondral tissue to lesion site was observed, and the same size a-cellular scaffolds (Hyalofast®) were cut and placed on the defects. Following the closure of joint capsule, single dose 1 ml TA was injected into the knee joints of group 1 (*n* = 9) and group 2 (*n* = 9) remained as a control group, respectively (Fig. 1). All surgical procedures were performed under sterile conditions and anesthesia. Anesthetic induction was obtained with Ketavet (Farmaceutici Gellini, Italy) and Xylazine (Rompun®, Bayer, Leverkusen, Germany) administration by intramuscular injection.

All animals received antibiotic prophylaxis of cephalosporin sodium (Ibrahim Etem Ulagay, Iespor®, Turkey) subcutaneously, and analgesia was achieved with deksketoprofen trometamol (Menarini, Arveles®, Italy). After the operation, rabbits were maintained in small cages for 2 weeks, a splint immobilized the knee joint for 10 days and then allowed to move freely. All animals (control and experimental group) were sacrificed under anesthesia of intravenous lethal injection of Tanax (Hoechst Veterinary GmbH, Germany) for the extraction of the condyles for histologic study at fourth (*n* = 8, 16 samples) and eighth (*n* = 10, 20 samples) weeks after surgery.

### Materials

Tranexamic acid (trans-4-amino-methylcyclohexane-1-carboxylic acid) is reversibly bound to the lysine portion of plasminogen to inhibit plasminogen activation [14]. It was determined that TA which is used in orthopedic surgery, prevents fibrin degradation, and has no negative effect on chondral cell viability [19, 20]. In the previous experimental studies, 10 mg/ml dose was given as the minimum effective dose for fibrin formation and did not affect chondrocyte cell viability, morphology, and extracellular matrix forming ability [20, 21].

Hyalofast is composed of a single three-dimensional fibrous layer of Hyaff®, a benzyl ester of HA, a natural component of the extracellular matrix. It can be cut and adaptively fit into irregular lesions aided by its soft and non-woven structure. Hyalofast can be implanted in mini-arthrotomy or arthroscopic surgery in any orientation or stacked due to its single uniform layer of Hyaff. Once implanted, it maintains its structure to support mesenchymal stem cell attachment, proliferation, and differentiation to completely fill the lesion. As Hyaff® degrades over time, it releases hyaluronic acid into the lesion and forming an embryonic-like microenvironment rich with HA [22].

### Histological and histomorphometric analysis

Macroscopic examination of the implantation site was performed by observing the appearance of the tissue in situ and by photographic documentation. Any abnormalities were recorded with details of the location, color, shape, and size, and grading was performed by using Brittberg scoring. From each implanted knee, the implantation site (femur cartilage at knee joint) was collected for hematoxylin-eosin and safranin-o staining. Histological evaluation of the administration site was performed using O’Driscoll scoring. Sections were independently scored by two investigators without knowledge of the study group being examined. Specimens were scored according to a histological grading scale which is a modification of that described by Driscoll et al. composed of five categories: cell morphology, matrix staining, surface regularity, the thickness of the cartilage, and bonding, with a total score range from 0 to 16 [23]. Grading was performed at a magnification of × 100.

### Statistical analysis

Data were organized in a Standart Statistical Package System (SPSS) v.22 software package (SPSS/PC Inc., Chicago, IL.) and reported as median (min-max) and mean. Repeated measures ANOVA test was used to compare groups and condyle effects together for each week. *p* values below 0.05 were considered as statistically significant.

## Results

In total, 18 subjects were enrolled in the study. None had any wound infection or death. Slight edema and swelling were observed in the knee joint regions of all subjects in the first 7 days. During the sacrification, all defect-forming areas were macroscopically distinguishable from the surrounding cartilage tissue and no synovitis or infective tissue was detected on the surgical site.

Macroscopic examination of tissue samples taken at fourth week postoperatively showed that both groups of lesions were filled with repair tissue. At the eighth week, all lesions were completely covered with regeneration tissue. Grade 2 nearly normal recovery was observed in three samples taken from group 1 in the fourth week; however, this improvement was not observed in any of the specimens in the group 2. In the control group, grade 4 severely abnormal level was seen in one specimen, while other samples were observed to be repaired with an abnormal level of grade 3. Grade 1 improvement was observed in one sample in group 1, and other samples were detected as grade 2 in the eighth week. On the other hand, in group 2, five samples were grade 2 and four samples were grade 3. In particular, the lesion site in the group 1 was more homogeneous (Additional file 1).

**Fig. 2:**
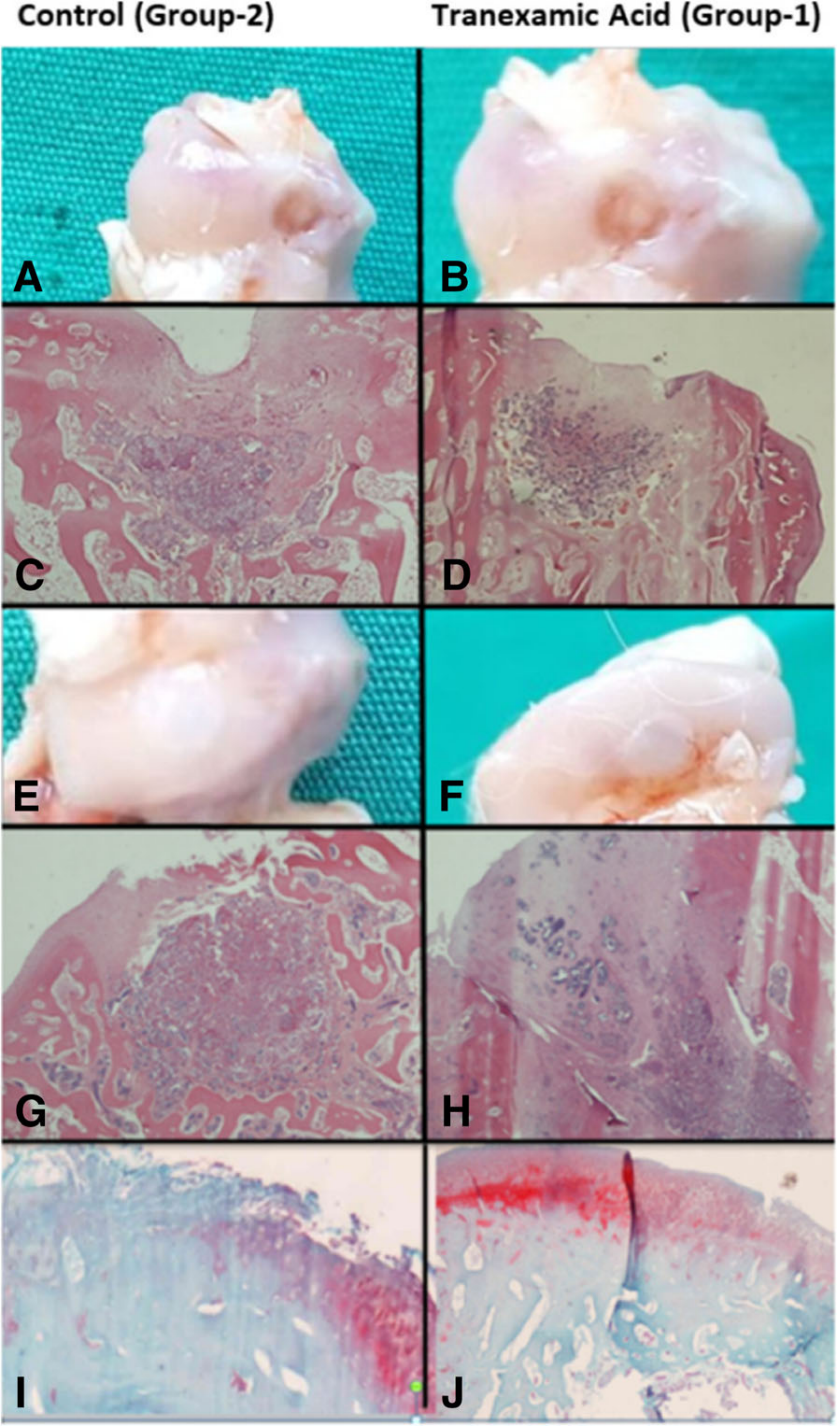
Macroscopic and microscopic views of the samples (control group: **a**, **c**, **e**, **g**, **i**; TA group: **b**, **d**, **f**, **h**, **j**). **a**, **b** Macroscopic views of samples at fourth week. **c**, **d** Microscopic views of samples at fourth week. **e**, **f** Macroscopic views of samples at eighth week. **g**, **h** Microscopic views of samples at eighth week. **i**, **j** Microscopic view of samples stained with safranin-o at eighth week

Histopathologically, it was found that the regeneration rate of subchondral tissue in the group 1 was higher when compared with group 2 at the fourth week. Moreover, the onset of cartilage tissue regeneration was observed in some subjects of group 1. Complete subchondral tissue regeneration was achieved in both groups at the end of eighth week. There was no original cartilage tissue regeneration in the groups. However, cartilage tissue repair rate and hyaline cartilage tissue ratio were found to be higher in group 1, when compared with group 2 at the eighth week (Fig. 2) (Table 1).

**Table 1:** Histologic scale which graded cartilage samples

Score		Fourth week		Eighth week	
		TA	Control	TA	Control
Cell morphology					
Hyaline cartilage	4			1	
Mostly hyaline cartilage	3			4	2
Hyaline and fibrocartilage	2	2		5	4
Mostly fibrocartilage	1	2	2		4
Mostly non-cartilage	0	4	6		
Matrix staining					
Same as the normal area	4				
Slightly reduced	3			6	
Reduced	2			4	
Significantly reduced	1	2			4
None	0	6	8		6
Surface regularity					
Smooth	2	2		6	5
Slightly Irregular	1	5	4	4	5
Irregular	0	1	4		
Thickness of the cartilage (%)					
100	4				
75	3			2	
50	2			5	
25	1	3		3	3
0	0	5	8		7
Bonding					
Both edges integrated	2	2		4	
One edge integrated	1	3	4	6	3
Both edges not integrated	0	3	4		7

### Statistical analysis

There were no statistically significant differences in the group and area interactions in the fourth week (*p* = 0.390). The site of specimen whether taken from medial or lateral condyle does not have an influence on the score values between the groups. Neither main influence of group nor condyle were found to be significant (*p* = 0.263) (Additional file 2). There were no statistically significant differences in the group and area interactions in the eighth week (*p* = 0.124). The site of specimen whether taken from medial or lateral condyle did not have an influence on the score values between the groups. Main condyle influence was found to be insignificant (*p* = 0.880) whether main group influence was found to be important (*p* = 0.002) with significantly increased values in the tranexamic acid group, both with lateral and medial condyle specimens when compared with the control group (Additional file 3).

No statistically significant difference was observed between the groups in the fourth week (*p* = 0,259), but in the eighth week, group 1 was significantly higher than group 2 (*p* = 0.002). The mean value of group 2 was measured as 4.2 ± 3.03 while the mean value of group 1 was 9.6 ± 3.58 for medial condyles. The mean value of group 2 was measured as 3.0 ± 1.41 while the mean of group 1 was 10.6 ± 1.34 for lateral condyles (Table 2).

**Table 2:** Mean and median O’ Driscoll scores of the two groups

			Control	Tranexamic acid	*p*
Fourth week	Medial	Mean ± SD Median (min-max)	1.50 ± 1.73 1.50 (0–3)	4.25 ± 4.35 4.50 (0–8)	0.263
	Lateral	Mean ± SD Median (min-max)	1.25 ± 1.50 1.00 (0–3)	2.50 ± 1.29 2.50 (1–4)	
	*p*		0.259		0.390
Eighth week	Medial	Mean ± SD Median (min-max)	4.20 ± 3.03 2.00 (2–8)	9.60 ± 3.58 7.00 (7–14)	0.880
	Lateral	Mean ± SD Median (min-max)	3.00 ± 1.41 2.00 (2–5)	10.60 ± 1.34 10.0 (9–12)	
	*p*		0.002		0.124

## Discussion

Osteochondral injuries are challenging clinical issues but also an area of interest in terms of recent material improvement and surgical innovations. Prior cartilage treatment researches revealed debridement, abrasion, and drilling methods while current improvements revised the definition of microfracture methods [24–26]. The initial description of the microfracture technique was reported by Stedman and colleagues in 1994, which is still in use for the treatment of osteochondral lesions with successful results of 11-year follow-ups [27, 28]. Microfracture technique can be considered as the first-line treatment for osteochondral lesions due to the cost-effectivity, single session application availability, and the short- and middle-term successful results of the method. However, the long-term efficacy of the method is still under debate.

A previous prospective study reported that 65% of young athletes under the age of 40 who underwent microfracture returned to sports, and this rate was found 20% in the elder patients [29]. After 2 years, the clinic worsened in all groups and the results were found to be worsened earlier, if the joint cartilage was exposed to early weight bearing. Gudas et al. demonstrated that surface fibrillation and fibrocartilage tissues were the dominant tissues in the areas of repair which were treated with microfracture technique in the 3-year follow-ups of young athletes [30].

Histological evaluation of experimental studies demonstrated a dominant fibrocartilage structure with a lesser hyaline cartilage feature in the regeneration tissue. The major contributor to this complex structure was found to be lack of an unavailable extracellular environment for the mesenchymal cells to differentiate and for the regeneration tissue to stabilize [29–31]. As a common determination of these studies, early weight bearing prior to the maintenance of appropriate regeneration tissue leads to a damage in cartilage healing. We aimed to maintain a more rapid and stabilized regeneration tissue to avoid poor outcomes.

Recently, three-dimensional cell implants with chondroconductive and osteoconductive properties produced from synthetic and biological materials by tissue engineers have a favorable clinical preference in order to prevent stabilization problems of the repaired tissue [31]. Among these, HA structured scaffolds which are bioavailable and bioabsorbable with a chemical structure suitable for the covalent binding of chondrocytes are favorable implants. Moreover, they facilitate collagen (types 1 and 2) production from mesenchymal stem cells via TGF that improves chondrogenic improvement [31]. Thus, we also preferred HA-structured a-cellular scaffolds in our study.

Demonstrating the benefits of HA structured scaffolds, Siclari et al. reported that 52 patients between the ages of 25 and 65 treated who were treated with microfracture and HA scaffold had significantly better clinical scores. Among these, the histological evaluations of the biopsies of 13 patients revealed a homogenous hyaline-like cartilage repair tissue [11]. A previous study of Gill et al. showed clinical healing of 87% on the osteochondral lesions with the 3.4 cm^2^ width after microfracture and scaffold implantation at the 27-month follow-up of 27 patients [24].

In the mid-term results of various clinical studies, the superiority of microfracture and scaffold application compared to microfracture is presented clinically and radiologically [32–34]. Erggelet et al. histologically reported the superiority of microfracture and cell-free scaffold application according to microfracture, and it was suggested to use microfracture together with scaffold in clinical applications in the damage of the full layer small cartilage defects [12]. Goyal et al. showed that younger patients and small lesions tend to have a better prognosis in the first 5 years, but osteoarthritis developed in all lesions after 5 years (30). Since microfracture and scaffold applications are shown to be insufficient in the formation of solid cartilage tissue against weight bearing on the knee joint [35], we planned to design this study.

Autologous chondrocyte implantation (ACI) has become prominent in recent years. In the study of Knutsen et al. in 2007, no significant difference was found between ACI and microfracture in 2 and 5 years follow-up retrospectively [36]. No significant difference was found between the ACI and microfracture in major chronic defects in the 14–15-year follow-up results of the same team [37, 38]. Although this method was reported to be the best treatment option in acute and large lesions of young active patients, its clinical use is limited due to it being a two-step surgery, high cost, not recommended to elderly patients, and with similar results according to microfracture in the early period of small defects.

More recently, TA is a popular molecule in orthopedic surgery which is frequently used for the prevention of perioperative and postoperative complications by decreasing the amount of bleeding during knee surgery [39]. Considering the mechanism of action, it is an effective molecule for the stabilization of fibrin [40]. In a previous study of Ambra and colloquies, no dose-dependent negative effect on cartilage viability was reported when TA was applied topically [19]. However, Parker and colloquies revealed that increasing the concentrations of TA above 20 mg/ml in vitro is associated with increased chondrocyte death. Suggesting the dose-dependent damage of TA, Turtle et al. showed that TA was found to be cytotoxic to bovine and murine cartilage at the dose of 100 mg/mL; however, the safety cutoff dose was found to be 25 mg/ml for TA [41, 42].

In our study, in order to form a more stable and fast fibrin clot in the regeneration area, TA was used intraarticularly, and more stable fibrin tissue formation and rapid regeneration were observed in microscopic and macroscopic evaluations. The mean scores obtained at the end of the fourth week were significantly higher in group 1, and it was observed that regeneration started more rapidly in the samples in this group, the repair tissue became more resistant to external forces in a shorter time, and cartilage repair started more rapidly in the eighth week.

Macroscopic results of our study revealed that the regularity of the surface was smoother and the tissue stability was more robust with higher mean Brittberg scores in group 1 when compared with group 2, both at the fourth and eighth weeks. Moreover, subchondral tissue and cartilage tissue regenerations were found to be more rapid and organized in group 1 without any negative effects on mesenchymal cells in microscopic evaluations. Suggesting our results, a previous experimental study reported that TA does not have a negative effect on chondrocyte viability and extracellular cell matrix production at appropriate doses in vitro [19].

## Conclusion

To conclude, in our study, we reported that TA clearly improved the healing of osteochondral defects which are treated by microfracture and a-cellular scaffold when applied intraarticularly with a significant improvement of healing time and quality of repair tissue. Thus, our findings were compatible with our hypothesis, stating that TA possibly improves the healing time and tissue stability in osteochondral defects which are treated by microfracture and a-cellular scaffold application. However, further long-term experimental and clinical studies are needed to demonstrate the original cartilage regeneration.

## Additional files

Additional file 1: Macroscopic scoring of the groups (Brittberg score). (PNG 15 kb)

Additional file 2: Microscopic scoring of the groups (O’Driscoll score) at fourth week. (PNG 12 kb)

Additional file 3: Microscopic scoring of the groups (O’Driscoll score) at eighth week. (PNG 12 kb)

## Abbreviations

ACI: Autologous chondrocyte implantation
AIBU: Abant Izzet Baysal University
HA: Hyaluronic acid
SPSS: Standart Statistical Package System
TA: Tranexamic acid
