# Supplementary figures and images for: Effects of tranexamic acid on the recovery of osteochondral defects treated by microfracture and acellular matrix scaffold: an experimental study
Source: J Orthop Surg Res. 2019 Apr 15;14:105. doi: 10.1186/s13018-019-1144-7 (PMC6469115; doi:10.1186/s13018-019-1144-7)

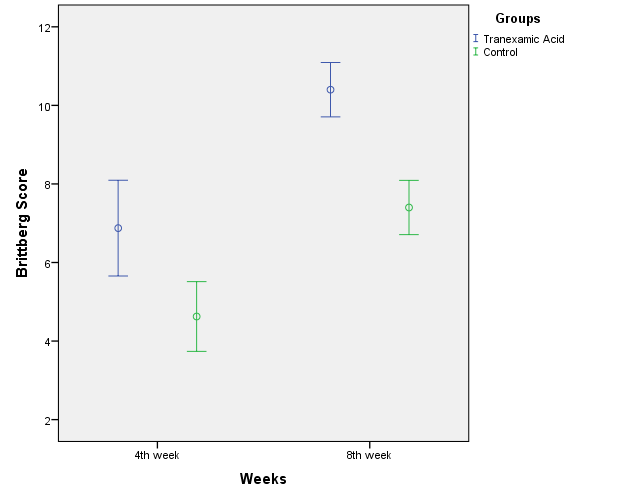

Supplement: Supplementary file 1 — Macroscopic scoring of the groups (Brittberg score). (PNG 15 kb) [file 13018_2019_1144_MOESM1_ESM.png]

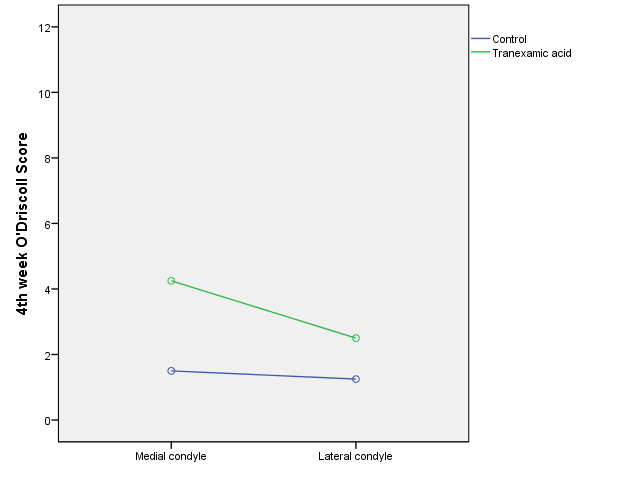

Supplement: Supplementary file 2 — Microscopic scoring of the groups (O’Driscoll score) at fourth week. (PNG 12 kb) [file 13018_2019_1144_MOESM2_ESM.png]

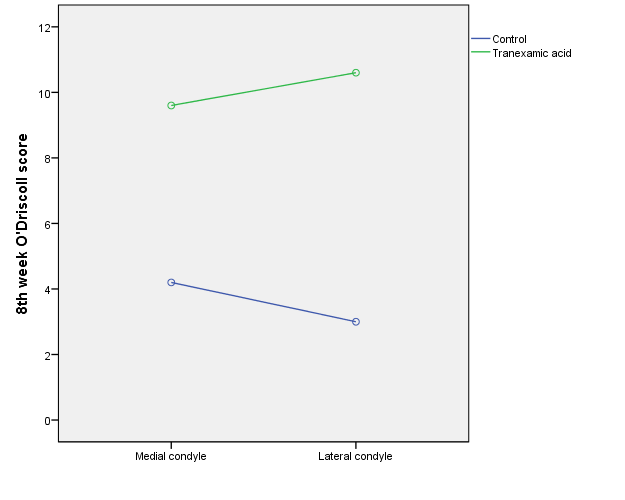

Supplement: Supplementary file 3 — Microscopic scoring of the groups (O’Driscoll score) at eighth week. (PNG 12 kb) [file 13018_2019_1144_MOESM3_ESM.png]
